# Insomnia as a Public Health Issue: Sociomedical Determinants in the Adult Population of Serbia

**DOI:** 10.3390/medicina62061098

**Published:** 2026-06-05

**Authors:** Nemanja Murić, Zoran Bukumirić, Maja Murić, Snežana Radovanović, Jovana Ristić, Danijela Djoković, Milan Djordjić, Vladimir Janjić

**Affiliations:** 1 Department of Psychiatry, Faculty of Medical Sciences, University of Kragujevac, 34000 Kragujevac, Serbia; nmuric91@gmail.com (N.M.); vladadok@yahoo.com (V.J.); 2 Clinic of Psychiatry, University Clinical Center Kragujevac, 34000 Kragujevac, Serbia; 3 Institute for Medical Statistics and Informatics, Medical Faculty, University of Belgrade, 11000 Belgrade, Serbia; zoran.bukumiric@med.bg.ac.rs; 4 Department of Physiology, Faculty of Medical Sciences, University of Kragujevac, 34000 Kragujevac, Serbia; majanikolickg90@gmail.com; 5 Center of Excellence for the Study of Redox Balance in Cardiovascular and Metabolic Disorders, University of Kragujevac, 34000 Kragujevac, Serbia; 6 Department of Social Medicine, Faculty of Medical Sciences, University of Kragujevac, 34000 Kragujevac, Serbia; jovanarad@yahoo.com; 7 Institute of Public Health Kragujevac, 34000 Kragujevac, Serbia; 8 Medical Faculty, University of Belgrade, 11000 Belgrade, Serbia; jovanaristic00@gmail.com; 9 Department of Communication Skills, Ethics and Psychology, Faculty of Medical Sciences, University of Kragujevac, 34000 Kragujevac, Serbia; mcpikac@gmail.com

**Keywords:** insomnia, prevalence, insomnia severity index, sociodemographic factors, comorbidities, psychosocial factors, lifestyle factors, Serbia

## Abstract

*Background/Objectives*: Insomnia is a prevalent sleep disorder with substantial public health implications, yet epidemiological data from Serbia remain limited. This study aimed to assess the prevalence of clinically significant insomnia symptoms in the adult population of Serbia and to examine associated sociodemographic, comorbidity, psychosocial, and lifestyle factors. *Materials and methods*: A cross-sectional study was conducted from September 2023 to September 2025, including 2577 adults aged 18–89 years across Serbia. Insomnia symptom severity was measured using the Insomnia Severity Index (ISI), with scores ≥ 15 indicating clinically significant insomnia symptoms. Sociodemographic, comorbidity, psychosocial, and lifestyle factors were assessed via self-reported questionnaires. Multivariable logistic regression with LASSO variable selection was used to identify factors independently associated with clinically significant insomnia symptoms. *Results*: The prevalence of clinically significant insomnia symptoms (ISI ≥ 15) was 10.9%. Independent factors associated with clinically significant insomnia symptoms included being single (OR = 1.54) or divorced (OR = 1.75), lower educational attainment (OR = 0.71 per level increase), being retired (OR = 1.83) or a student (OR = 1.66), dermatological comorbidities (OR = 2.99), use of anxiolytic medications (OR = 2.44), exposure to stressful life events (OR = 1.88), engagement in late-night activities (OR = 1.37), consumption of coffee/tea (OR = 2.22), energy drink consumption (OR = 1.52), and late-night eating habits (OR = 1.27). *Conclusions*: Clinically significant insomnia symptoms among adults in Serbia are influenced by a complex interplay of sociodemographic, comorbidity, psychosocial, and lifestyle factors. These findings underscore the need for integrated approaches that address both medical and modifiable behavioral determinants in the prevention and management of insomnia symptoms.

## 1. Introduction

Insomnia is the most prevalent sleep disorder and is consistently linked to a wide spectrum of adverse outcomes, including psychosomatic conditions and reduced quality of life [1]. Beyond its clinical manifestations, insomnia often leads to substantial impairment in daily functioning and social interactions, which is a primary concern for affected individuals and frequently motivates them to seek professional help. However, only about half of insomnia patients seek and reach specialized treatment [2]. Daytime impairments in patients with insomnia involve daytime fatigue, cognitive dysfunction, and mood instability as core symptoms [3], which collectively contribute to higher rates of workplace injuries and traffic accidents [4]. Furthermore, insomnia is associated with a substantial economic burden on healthcare systems, employers, and society, driven by increased healthcare utilization, reduced workplace productivity, and elevated accident risk [5].

According to the Diagnostic and Statistical Manual of Mental Disorders, Fifth Edition (DSM-5), insomnia is defined as persistent difficulty initiating or maintaining sleep or experiencing early-morning awakenings, occurring at least three times per week for a minimum duration of three months despite adequate sleep opportunities. Furthermore, these symptoms must not be attributable to other medical, psychiatric, or substance-related conditions [6]. In epidemiological research, however, insomnia is often assessed using validated self-report instruments rather than clinical interviews [7].

Globally, the prevalence of insomnia ranges from 10% to 30% of the population [8]. Recent studies report that 16.2% of adults aged ≥20 years experience clinically significant insomnia symptoms, nearly half of whom report severe symptoms [9]. Such variability in prevalence may be explained by limited public awareness of insomnia and the complexity of diagnostic criteria required for a definitive diagnosis [5].

European data further highlight this variability. Surveys conducted in France, Germany, Italy, Spain, and the United Kingdom have reported prevalence rates of approximately 6% [10], whereas other studies from France have found substantially higher prevalence rates exceeding 30% [11]. These findings underscore the importance of harmonized diagnostic approaches and increased awareness to better capture the true burden of insomnia across different populations. In the Western Balkans, the prevalence of sleep disorders appears to be consistent with global estimates. A study conducted in Croatia reported that more than half of participants experienced sleep problems, with approximately 30% reporting difficulties daily or several times per week. These sleep disturbances were particularly common among older adults and women [12]. Another study conducted during the third wave of the Coronavirus Disease 2019 (COVID-19) pandemic found that approximately half of young adults from this region reported disrupted sleep patterns [13].

There is increasing recognition that insomnia arises from complex interactions between environmental, behavioral, and biological factors rather than from a single cause [14]. Previous studies have identified associations between insomnia and various sociodemographic and health-related factors, with higher prevalence observed among women [9], older adults [15], individuals with chronic diseases or poor health status [16], and those with lower educational attainment, socioeconomic disadvantage, or residence in rural areas with limited access to healthcare [15]. Moreover, insomnia has been linked to increased cardiometabolic risk, including hypertension and type 2 diabetes mellitus, as well as elevated morbidity and mortality from cardiovascular and cerebrovascular diseases [17]. However, the interplay of these factors within specific populations remains incompletely understood.

Despite these findings, comprehensive epidemiological studies examining clinically significant insomnia symptoms based on the Insomnia Severity Index (ISI) in the adult population of Serbia remain scarce, particularly with regard to associated sociodemographic and health-related factors. Identifying and addressing vulnerable subgroups may facilitate the development of more effective preventive measures and targeted therapeutic strategies for individuals experiencing sleep disturbances. Therefore, the present study aimed to assess the prevalence of clinically significant insomnia symptoms using the ISI in a large adult population in Serbia and to examine their associations with sociodemographic characteristics, health-related factors, and lifestyle behaviors.

## 2. Materials and Methods

### 2.1. Ethical Considerations

This study was approved by the Ethics Committee of the University Clinical Center of Kragujevac, Serbia (Approval No.: 01-20-186, Date of Approval: 11 February 2020). The study was performed in accordance with the principles of the Declaration of Helsinki and relevant national regulations governing research involving human participants. All participants voluntarily provided written informed consent prior to participation in the study.

### 2.2. Study Design

This cross-sectional, observational study was conducted between 1 September 2023 and 1 September 2025 and included 2577 adults aged 18 to 89 years from the Republic of Serbia. Although the study was initially planned to begin in 2020 following ethical approval, its initiation was interrupted by the COVID-19 pandemic, and the data collection was postponed until after the end of the pandemic to minimize its potential influence on the results. Eligibility for participation was determined using predefined inclusion and exclusion criteria. The inclusion criteria were (1) age ≥ 18 years; (2) absence of diagnosed somatic illnesses or the presence of chronic somatic conditions in a stable phase, with stable therapy for at least three months prior to completion of the study instruments; and (3) ability to understand the nature of the study and provision of written informed consent. The exclusion criteria included (1) age below 18 years of age; (2) inability to understand the study procedures or illiteracy; (3) self-reported history of mental disorder(s), previously diagnosed by a psychiatrist and/or dependence on psychoactive substances, assessed using questionnaires administered in this study.

### 2.3. Sampling and Recruitment

**Figure 1:**
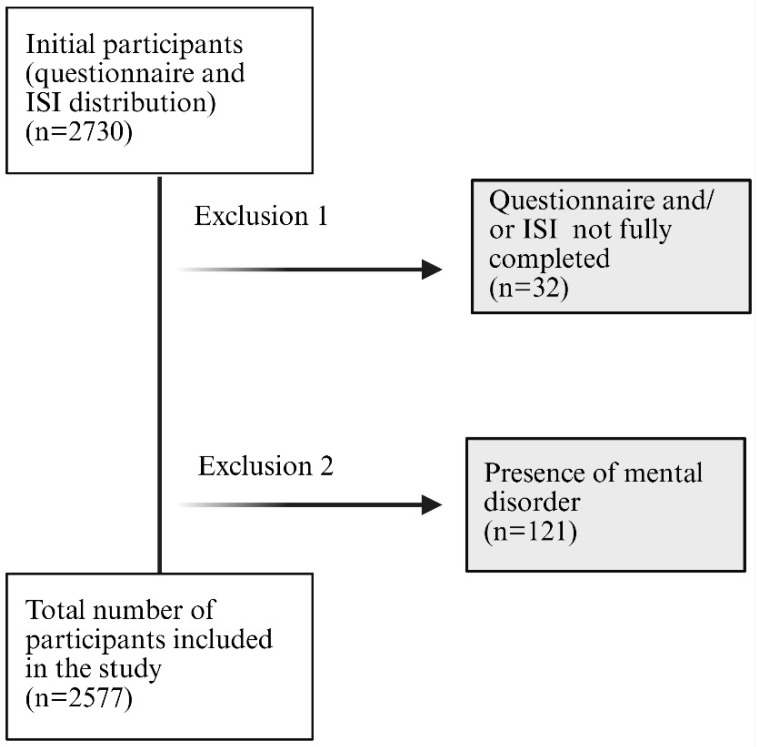
Flowchart of participant screening and inclusion in the study.

Participants were recruited across all regions of Serbia (Vojvodina, Belgrade region, Šumadija and Western Serbia, and Southern and Eastern Serbia) through collaboration with local community offices within municipalities. In each region, paper-based questionnaires were randomly distributed to residents of selected municipalities. The survey included a questionnaire on sociodemographic characteristics and health-related factors, and the ISI questionnaire. Participants were instructed to complete all items independently. The screening process, the reasons for participant exclusions, and the final sample included in the study are presented in Figure 1.

### 2.4. Questionnaires

A general questionnaire assessing sociodemographic characteristics, health-related factors, and lifestyle behaviors was specifically developed for the purposes of this study. The design of the questionnaire was guided by previous studies investigating sociodemographic and lifestyle factors associated with sleep disorders [18]. The questionnaire collected information on participants’ age, sex, marital status, children, educational attainment, employment status, region of residence, type of residence and living environment. In addition, the questionnaire included questions regarding preexisting comorbidities, psychiatric heredity, use of psychotropic medications, and exposure to stressful life events during the previous month. Lifestyle behaviors were also assessed, including physical activity, engagement in late-night activities (e.g., watching television, reading, or hobbies), cigarette smoking, alcohol consumption, consumption of coffee/tea and energy drinks, and dietary habits. Most variables were assessed using predefined categorical response options.

### 2.5. Insomnia Severity Index (ISI)

The severity of insomnia symptoms was assessed using the Insomnia Severity Index (ISI), a widely used self-report instrument. Although the ISI has not yet undergone formal psychometric validation in the Serbian population, an official Serbian translation was used in this study, which has previously been extensively used in Serbian research, including academic and doctoral research projects. The ISI consists of seven items evaluating difficulties related to sleep initiation, sleep maintenance, early morning awakening, satisfaction with sleep, interference with daily functioning, noticeability of sleep problems, and distress caused by sleep difficulties. Each item is rated on a five-point Likert scale ranging from 0 (no problem) to 4 (very severe problem). The total ISI score is calculated by summing the responses to all items, resulting in a total score ranging from 0 to 28. Based on the total score, insomnia severity can be categorized as follows: no insomnia (0–7), subthreshold insomnia (8–14), moderate severity of insomnia symptoms (15–21), and severe insomnia symptoms (22–28) [19]. In this study, ISI scores ≥ 15 were used to define clinically significant insomnia symptoms.

### 2.6. Statistical Analysis

Data analysis was performed using IBM SPSS Statistics for Windows, version 26.0 (IBM Corp., Armonk, NY, USA). LASSO regression was conducted in R version 4.5 (R Foundation for Statistical Computing, Vienna, Austria) using RStudio version 2024.12.1 (Posit Software, PBC, Boston, MA, USA). Descriptive and analytical statistical methods were employed in the analysis. Continuous variables with a normal distribution were expressed as mean ± standard deviation, whereas those with a nonparametric distribution were reported as median and range (min–max). Categorical variables were presented as absolute frequencies and percentages. Differences in the distribution of independent variables across categories of the dependent variable were assessed using Pearson’s chi-square test or Fisher’s exact test for categorical data. For continuous variables, comparisons were performed using Student’s *t*-test in the case of a normal distribution and the Mann–Whitney U test for nonparametric data. To identify factors associated with the outcome, logistic regression analysis was conducted. The set of candidate variables for the multivariable logistic regression model included those that were statistically significant in univariable analyses (*p* < 0.05), as well as variables identified in the literature as clinically relevant associated factors of the outcome. Variable selection was performed using LASSO (Least Absolute Shrinkage and Selection Operator) regularization, which enables simultaneous variable selection and coefficient shrinkage. The optimal penalty parameter (λ) was determined using cross-validation, resulting in a final model that retained only the most informative factors. Statistical significance was set at *p* < 0.05 for all analyses.

## 3. Results

### 3.1. Sociodemographic Characteristics According to Insomnia Status

Table 1 presents the sociodemographic characteristics of the study population according to insomnia status. A total of 2577 participants were included, of whom 281 (10.9%) had clinically significant insomnia symptoms (ISI score ≥ 15), while 2296 (89.1%) did not meet the criteria for clinically significant insomnia symptoms (ISI score < 15).

No statistically significant difference in sex distribution was observed between participants with and without clinically significant insomnia symptoms (*p* = 0.259). Males accounted for 52.0% of participants with clinically significant insomnia symptoms and 48.4% of those without clinically significant insomnia symptoms, while females represented 48.0% and 51.6% of the groups, respectively. The median age in the total sample was 39 years (range 18–89). Participants with clinically significant insomnia symptoms were slightly younger (35 years) compared with those without clinically significant insomnia symptoms (39 years), although the difference did not reach statistical significance (*p* = 0.052). However, age group distribution differed significantly between the two groups (*p* = 0.043). Clinically significant insomnia symptoms were most prevalent among younger adults aged 18–29 years (33.8%), whereas participants without clinically significant insomnia symptoms were more frequently represented in the 30–44 (37.9%) and 45–59 (26.4%) age groups.

Marital status was significantly associated with the presence of clinically significant insomnia symptoms (*p* < 0.001). Participants with clinically significant insomnia symptoms were more frequently single or not married (48.0%) compared with those without clinically significant insomnia symptoms (38.6%). In contrast, married individuals were more common among participants without clinically significant insomnia symptoms (51.3%) than among those with clinically significant insomnia symptoms (37.7%). Divorced and widowed individuals were also slightly more represented in the group of participants with clinically significant insomnia symptoms. No statistically significant difference was found regarding having children (*p* = 0.956), with similar proportions of participants with and without children in both groups.

Regional distribution showed a statistically significant difference (*p* = 0.010). Participants with clinically significant insomnia symptoms were more frequently from Šumadija and Western Serbia (31.0%), while those without clinically significant insomnia symptoms were more commonly from Vojvodina (29.3%) and the Belgrade region (25.0%). No significant differences were observed with regard to living environment (urban vs. rural) (*p* = 0.850) or residential type (house vs. apartment) (*p* = 0.159).

A statistically significant difference in educational attainment was observed between the analyzed groups (*p* < 0.001). Overall, the largest proportion of participants had a university/PhD degree (44.3%), followed by those with secondary school education (40.9%). Post-secondary/college education was reported by 12.7% of participants, while only 2.1% had completed primary school. Participants with secondary school education were more prone to clinically significant insomnia symptoms (52.5%), followed by university/PhD education (35.4%). A smaller proportion of participants had post-secondary/college education (8.2%), while primary school education was reported by 3.9% of individuals. The distribution among participants without clinically significant insomnia symptoms was similar to that observed in the total sample, with the majority of participants having university/PhD education (45.4%), followed by secondary school education (39.5%). Post-secondary/college education accounted for 13.2% of participants, while primary school education was the least represented (1.9%).

Employment status demonstrated a strong association with the presence of clinically significant insomnia symptoms (*p* < 0.001). Students (12.5%) and retired individuals (16.7%) were more frequently represented among participants with clinically significant insomnia symptoms compared to those without clinically significant insomnia symptoms, whereas employed individuals were less likely to report clinically significant insomnia symptoms (66.5% vs. 77.8%).

### 3.2. Comorbidities, Medication Use, and Psychosocial Factors Associated with Insomnia

Table 2 presents the distribution of comorbidities, medication use, psychiatric heredity, and stressful life events in the past month according to insomnia status. Participants with clinically significant insomnia symptoms more frequently reported the presence of any comorbidity compared with those without clinically significant insomnia symptoms (24.9% vs. 17.6%, *p* = 0.003). Among specific comorbidities, cardiovascular diseases were significantly more common in the group with clinically significant insomnia symptoms (18.9% vs. 10.7%, *p* < 0.001). Respiratory comorbidities were also more prevalent among participants with clinically significant insomnia symptoms (3.9% vs. 1.3%, *p* = 0.003). Similarly, oncological (1.8% vs. 0.6%, *p* = 0.048) and dermatological comorbidities (2.8% vs. 0.9%, *p* = 0.008) were reported more frequently among participants with clinically significant insomnia symptoms. In contrast, no significant differences were observed for rheumatological (*p* = 0.114), endocrine/metabolic (*p* = 0.473), gastrointestinal (*p* = 1.000), neurological (*p* = 1.000), or nephrological comorbidities (*p* = 1.000).

The use of psychotropic medications was significantly higher among participants with clinically significant insomnia symptoms (14.6% vs. 5.6%, *p* < 0.001). A similar pattern was observed for anxiolytic agents, which were more frequently used by individuals with clinically significant insomnia symptoms compared with those without clinically significant insomnia symptoms (13.5% vs. 5.2%, *p* < 0.001). No significant difference was found in the use of antidepressants (*p* = 0.095) or antiepileptic agents (*p* = 1.000). Psychiatric heredity was significantly less frequently reported in participants with clinically significant insomnia symptoms compared with those without clinically significant insomnia symptoms (2.5% vs. 6.9%, *p* = 0.003). In contrast, stressful life events were significantly more common among participants with clinically significant insomnia symptoms (48.8%) compared with those without clinically significant insomnia symptoms (32.7%) (*p* < 0.001).

### 3.3. Lifestyle Factors Associated with Insomnia

Table 3 presents lifestyle characteristics of the study population according to insomnia status. No significant difference was observed between participants with and without clinically significant insomnia symptoms regarding physical activity (*p* = 0.867), smoking (*p* = 0.783), or alcohol consumption (*p* = 0.460).

Several lifestyle-related variables were significantly associated with the presence of clinically significant insomnia symptoms. Participants with clinically significant insomnia symptoms more frequently reported engagement in activities in late hours, such as watching television, reading, or other hobbies (58.0% vs. 42.3%, *p* < 0.001). Consumption of coffee or tea was also significantly more common among participants with clinically significant insomnia symptoms (92.2%) compared with those without insomnia (84.3%) (*p* < 0.001). Similarly, energy drink consumption was significantly higher in the group with clinically significant insomnia symptoms, particularly daily consumption (5.7% vs. 1.1%, *p* < 0.001).

Dietary habits differed significantly between groups (*p* = 0.001), with irregular eating patterns being more frequent among participants with clinically significant insomnia symptoms compared with those without clinically significant insomnia symptoms (42.0% vs. 32.4%). In addition, late-night food consumption was significantly more common among individuals with clinically significant insomnia symptoms (*p* = 0.001), particularly eating sometimes 2–4 times per week (34.2% vs. 29.1%) or every day (26.7% vs. 18.9%).

### 3.4. Factors Associated with Insomnia: Multivariate Logistic Regression Analysis

**Figure 2:**
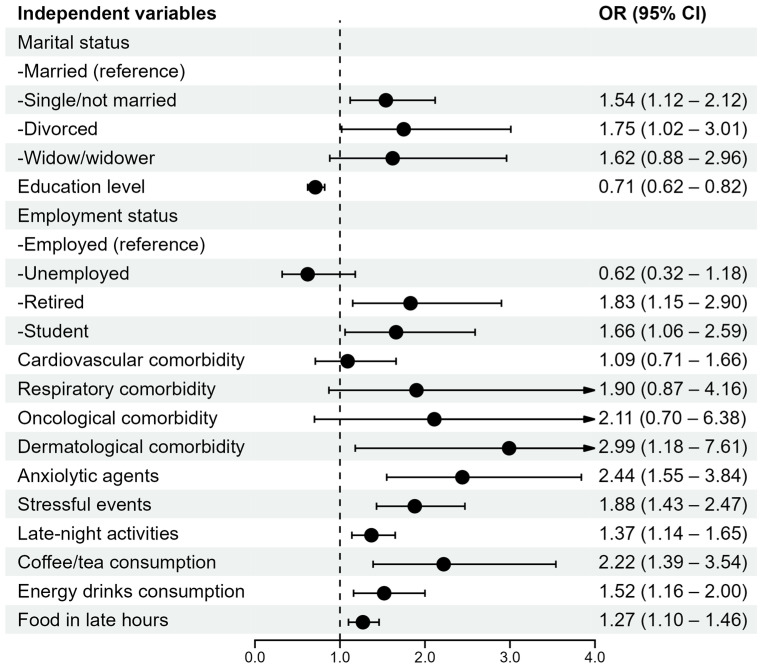
Graphic presentation of multivariable logistic regression analysis of factors associated with clinically significant insomnia symptoms. Black dots represent odds ratios (ORs), horizontal lines indicate 95% confidence intervals (95% CIs), and the vertical dashed line represents the null value (OR = 1).

The final multivariate logistic regression model included 13 associated factors and was performed on 2296 participants, of whom 280 had clinically significant insomnia symptoms. Variables showing multicollinearity were excluded from the final model. The overall model was statistically significant (*p* < 0.001) (Figure 2).

In the multivariable logistic regression model, the statistically significant factors associated with the presence of clinically significant insomnia symptoms were marital status, educational attainment, employment status, dermatological comorbidities, anxiolytic medications, stressful life events, late-night activities (TV watching, reading, hobbies), consumption of coffee or tea, consumption of energy drinks, and late-night eating habits.

Marital status was significantly associated with clinically significant insomnia symptoms. Compared with married participants as reference category, single individuals had a 54% higher odds of clinically significant insomnia symptoms (OR = 1.54; 95% CI: 1.12–2.12; *p* = 0.008), while divorced participants had 75% higher odds of clinically significant insomnia symptoms (OR = 1.75; 95% CI: 1.02–3.01; *p* = 0.042), in the adjusted model.

Educational attainment was inversely associated with the presence of clinically significant insomnia symptoms (OR = 0.71; 95% CI: 0.62–0.82; *p* < 0.001), indicating that higher educational attainment was associated with lower odds for clinically significant insomnia symptoms. In the adjusted model, each higher level of educational attainment was associated with approximately 30% lower odds of clinically significant insomnia symptoms.

Employment status was also a significant factor. Compared with employed individuals as the reference category, retired participants had 83% higher odds of clinically significant insomnia symptoms (OR = 1.83; 95% CI: 1.15–2.90; *p* = 0.010), while students had 66% higher odds of clinically significant insomnia symptoms (OR = 1.66; 95% CI: 1.06–2.59; *p* = 0.025), after adjustment. On the other hand, unemployment was not significantly associated with clinically significant insomnia symptoms.

Among comorbidities, only dermatological comorbidities were independently associated with clinically significant insomnia symptoms (OR = 2.99; 95% CI: 1.18–7.61; *p* = 0.021), with nearly three times higher odds of clinically significant insomnia symptoms in the adjusted model. Cardiovascular, respiratory, and oncological comorbidities were not significantly associated with the presence of clinically significant insomnia symptoms in the adjusted model.

Several lifestyle factors were independently associated with the presence of clinically significant insomnia symptoms. More frequent engagement in late-night activities (TV watching, reading, hobbies) was associated with 37% higher odds of clinically significant insomnia symptoms (OR = 1.37; 95% CI: 1.14–1.65; *p* = 0.001) in the adjusted model. Consumption of coffee or tea was also significantly associated with clinically significant insomnia symptoms (OR = 2.22; 95% CI: 1.39–3.54; *p* = 0.001). Participants who consume coffee or tea had 2.2 times higher odds of clinically significant insomnia symptoms, after adjustment. Similarly, higher consumption of energy drinks increased the odds of clinically significant insomnia symptoms by over 50% (OR = 1.52; 95% CI: 1.16–2.00; *p* = 0.002) in the adjusted model. Finally, late-night eating habits were significantly associated with clinically significant insomnia symptoms (OR = 1.27; 95% CI: 1.10–1.46; *p* = 0.001), indicating that more frequent food consumption in late evening hours increased the risk of clinically significant insomnia symptoms by 27% in the adjusted model.

## 4. Discussion

The present study provides a comprehensive analysis of sociodemographic, comorbidity, psychosocial, and lifestyle factors associated with clinically significant insomnia symptoms in a large adult population of Serbia. The prevalence of clinically significant insomnia symptoms (ISI ≥ 15) in our sample (10.9%) is consistent with epidemiological data indicating that DSM-defined insomnia affects approximately 12% of the general population [1]. However, a global individual participant data meta-analysis estimated that around 16% of adults have clinically relevant insomnia symptomatology based on the ISI, with moderate and severe symptoms observed across populations [20], which is slightly higher than our findings. Interestingly, European community studies reported even higher insomnia prevalence of 21.1%, with clinical insomnia present in 6.9% of the population. Furthermore, higher rates were observed in women and individuals exposed to psychosocial stressors [21]. In older adult cohorts, clinically significant insomnia rates have been reported at around 20%, supporting the notion that insomnia is a common condition in adult populations when assessed by validated scales like the ISI [22]. In this study, clinically significant insomnia symptoms were more prevalent among younger adults, particularly those aged 18–29 years. This finding contrasts with traditional views that insomnia increases with age but aligns with more recent evidence suggesting a growing burden of sleep disturbances among younger populations [23,24]. Contemporary studies indicate that insomnia in younger adults is increasingly driven by behavioral and lifestyle factors, including excessive digital media use, irregular sleep timing, and bedtime procrastination behaviors [24]. Recent systematic reviews confirm that screen exposure and social media engagement are consistently associated with poorer sleep quality and higher insomnia symptom severity in late adolescents and young adults [25]. Moreover, bedtime procrastination has emerged as a key behavioral mechanism influencing sleep outcomes, and it is particularly associated with reduced sleep sufficiency and increased daytime fatigue among young adults [26]. These patterns are further reinforced by performance goal orientation among students, with performance approach goals associated with lower bedtime procrastination and performance avoidance goals associated with higher bedtime procrastination, while academic stress and self-control act as key mediating mechanisms linking goal orientation to bedtime procrastination [27]. Collectively, these findings suggest that modern behavioral patterns play a central role in the rising burden of insomnia-related symptoms among younger populations.

Regional studies from the Western Balkans similarly report high rates of sleep disturbances. For example, a study conducted during the COVID-19 pandemic found that over half of young adults in Croatia and Bosnia and Herzegovina experienced disrupted sleep patterns, with 54.2% reporting subthreshold or more severe insomnia [12]. In Serbian student populations, poor sleep quality was frequently associated with psychological distress, highlighting the substantial burden of sleep problems in our country [28]. These findings place the current study within the broader Balkan context and underscore the need for region-specific public health strategies.

In our study, regional differences in the prevalence of clinically significant insomnia symptoms were observed, with participants from Šumadija and Western Serbia more frequently experiencing clinically significant insomnia symptoms compared with those from other regions. This statistically significant variation may reflect underlying sociodemographic, cultural, or healthcare-related disparities across regions. For example, the Belgrade region contributes the largest share of the national GDP, followed by Vojvodina, while Šumadija and Western Serbia account for a considerably smaller proportion, highlighting the lower economic output of this region [29]. Such economic and healthcare inequalities may increase psychosocial stress and reduce access to sleep-related healthcare, thereby contributing to the higher burden of insomnia symptomatology. Interestingly, no significant differences were observed with regard to living environment (urban vs. rural) or residential type (house vs. apartment). Previous studies have reported mixed results regarding urban–rural differences in sleep disturbances, with some identifying higher prevalence in urban areas [30], while other studies have shown opposite results [14]. Our findings may indicate that regional disparities in the presence of clinically significant insomnia symptoms are more likely driven by macro-level economic and healthcare inequalities than by micro-level residential characteristics. This highlights the importance of considering regional context and structural determinants when addressing insomnia in public health strategies, rather than focusing solely on individual living environments.

Our findings highlight the multifactorial nature of insomnia, with sociodemographic characteristics, comorbidities, psychosocial stressors, and lifestyle behaviors all contributing to the occurrence of clinically significant insomnia symptoms. Marital status emerged as a significant factor associated with clinically significant insomnia symptoms, with single and divorced individuals demonstrating higher odds compared with married participants. This is consistent with previous studies suggesting that social support and stable relationships may play a protective role in sleep quality [31]. Employment status also influenced the risk of clinically significant insomnia symptoms, with retired individuals and students demonstrating higher odds of clinically significant insomnia symptoms than employed participants. Prior studies have shown that insomnia symptoms are associated with earlier retirement, which is particularly related to poor health or disability [32]. Besides lower health status, these associations in our study may reflect lifestyle patterns, altered daily routines, or increased stress levels in these participants. Consistent with our results, a recent systematic review and meta-analysis of 34 studies demonstrated moderate associations between stress and both poor sleep quality and insomnia in undergraduate students, underscoring the vulnerability of this population to stress-related sleep disturbances [33]. In particular, academic stress has been identified as a key mediator linking bedtime procrastination and sleep disruption [27]. In accordance with that, the present findings indicate that educational attainment was inversely associated with clinically significant insomnia symptoms, with each increase in educational level associated with an approximately 30% reduction in the odds of clinically significant insomnia symptoms. This finding is consistent with prior evidence linking education to better sleep patterns [34] and health literacy [35].

Interestingly, in our multivariable analysis, age and sex were not independently associated with clinically significant insomnia symptoms. This contrasts with prior studies that consistently report higher prevalence among women (8) and older adults (14). Several explanations may account for this discrepancy. First, cultural and social factors specific to the Serbian population may attenuate the influence of age and sex, with other sociodemographic variables (e.g., marital status, employment, education) exerting stronger effects. Second, sample characteristics may have played a role: our study included a broad adult population rather than focusing on older cohorts, which may have diluted age-related differences. Third, confounding variables such as psychosocial stressors, comorbidities, and lifestyle behaviors may mediate the relationship between age, sex, and insomnia, thereby reducing their independent contribution in adjusted models. Finally, methodological differences, including the use of self-report instruments rather than clinical interviews or objective sleep measures, may also explain the divergence from previous findings. These considerations highlight the importance of examining insomnia symptomatology within specific cultural and epidemiological contexts, as risk factors may vary across populations.

Among medical comorbidities, only dermatological conditions were independently associated with increased risk for clinically significant insomnia symptoms. Participants with dermatological comorbidities had nearly three times higher odds of clinically significant insomnia symptoms, potentially due to discomfort, pruritus, or pain disrupting sleep. Inflammatory skin conditions frequently impair sleep, with nocturnal pruritus emerging as a primary mediator of sleep disturbance in atopic dermatitis and urticaria, contributing substantially to insomnia symptoms [36]. This finding may also support the growing recognition of bidirectional interactions between sleep regulation, inflammatory pathways, and chronic skin disease. Cardiovascular, respiratory, and oncological comorbidities were not significantly associated with clinically significant insomnia symptoms in the adjusted model, suggesting that the direct effects of these conditions on sleep may be mediated by other factors such as medication use or lifestyle behaviors. Use of anxiolytic medications was strongly associated with clinically significant insomnia symptoms, with a 2.4-fold increased odds of developing this symptomatology. While these medications are typically prescribed to manage anxiety or sleep disturbances, their use may reflect underlying anxiety disorders, which themselves contribute to sleep disruption. Stressful life events in the past month were also independently associated with clinically significant insomnia symptoms, with exposure increasing the odds by 88%, reinforcing the well-documented role of psychosocial stress as a precipitating and perpetuating factor for insomnia [37]. Psychosocial stressors, including stressful life events, may precipitate or exacerbate insomnia via heightened sleep reactivity and stress responses. Evidence from longitudinal studies demonstrates that individuals with greater stress reactivity and poorer sleep efficiency following stress are more likely to develop persistent insomnia over time [38].

Behavioral and lifestyle factors demonstrated significant associations with clinically significant insomnia symptoms. Engagement in late-night activities, including TV watching, reading, or hobbies, increased insomnia risk by 37%, likely reflecting delayed sleep onset or irregular sleep schedules. Habitual consumption of caffeine has been associated with greater difficulty falling asleep, shorter sleep duration, and increased insomnia symptoms in population-based studies, particularly among individuals with age-related sensitivity to caffeine’s effects [39]. In our study, consumption of coffee and/or tea doubled the odds of clinically significant insomnia symptoms, while higher intake of energy drinks increased the odds by 52%, consistent with the stimulating effects of caffeine on sleep quantity and quality [40]. However, reverse causality cannot be excluded, as individuals with insomnia symptoms may consume higher amounts of caffeine to counteract daytime fatigue and sleepiness. Additionally, late-night eating was associated with a 27% higher risk of clinically significant insomnia symptoms, potentially due to gastrointestinal discomfort, altered metabolism, or circadian rhythm disruption.

These findings have important implications for clinical practice and public health. First, interventions targeting modifiable lifestyle factors, such as limiting late-night screen time, reducing caffeine and energy drink intake, and improving sleep hygiene, may be effective strategies to mitigate clinically significant insomnia symptomatology. In addition, more structured behavioral recommendations could be implemented in routine health education, including establishing consistent sleep–wake schedules, avoiding stimulating activities in the hour before bedtime, and reducing digital media engagement during nighttime hours, particularly in younger populations. Given the strong association between late-night activities and clinically significant insomnia symptoms in our multivariable model, these behaviors should be specifically addressed as key intervention targets rather than general lifestyle advice. Second, identifying individuals with dermatological conditions, high stress levels, or those using anxiolytic medications could facilitate early screening and targeted support. Primary care providers and dermatologists could play an important role in opportunistic screening for insomnia symptoms using brief validated tools such as the ISI, enabling earlier recognition of patients at risk for chronic sleep disturbances. Particular attention should be given to patients presenting with stress-related complaints or frequent use of anxiolytic medication, as these groups demonstrated consistently elevated odds of clinically significant insomnia in the adjusted analysis. Third, public health initiatives aimed at increasing awareness of clinically significant insomnia symptoms and promoting healthy sleep behaviors, particularly among younger adults, single or divorced individuals, and students or retirees, may help reduce the burden of sleep disturbances in the population. These initiatives may be further strengthened through university-based sleep health programs, workplace wellness interventions, and the integration of digital cognitive behavioral therapy for insomnia (CBT-I) as a scalable, evidence-based treatment option accessible to high-risk groups. Importantly, interventions should be tailored to sociodemographic profiles identified in this study, including individuals with lower educational attainment and those residing in regions with higher insomnia prevalence (Šumadija and Western Serbia), suggesting the need for region-specific public health planning rather than uniform national strategies. Finally, given the strong independent associations observed with dietary behaviors (coffee/tea, energy drinks, and late-night eating), workplace and educational policies could incorporate sleep-health promotion strategies such as limiting access to energy drinks in academic settings and integrating sleep education into student health services.

### Limitations of the Study

Several limitations should be considered when interpreting the findings of this study. First, the cross-sectional design precludes the establishment of causal relationships between clinically significant insomnia symptoms and the associated sociodemographic characteristics, health-related and lifestyle factors. Therefore, the identified associations should be interpreted with caution. In addition, the differing reference timeframes across measures (e.g., ISI referring to the past two weeks vs. stressful life events to the past month) may introduce temporal misclassification and potential reverse causality. Second, although the study included a large sample of adult participants, all respondents were recruited within the Republic of Serbia, and the sample may overrepresent urban residents and individuals with higher educational attainment. This may limit the generalizability of the findings to populations with different cultural, socioeconomic, and healthcare contexts. Furthermore, the recruitment approach was based on field and community sampling procedures rather than probability-based random sampling, which may introduce selection bias. Third, the study relied on self-reported data, which may be subject to recall bias and social desirability bias, particularly for lifestyle-related behaviors such as caffeine consumption, alcohol intake, smoking, and stressful life events. Fourth, insomnia symptoms were assessed using the ISI, a validated self-report instrument, rather than clinical diagnostic interviews or objective sleep measures such as polysomnography or actigraphy. Consequently, misclassification is possible, and the results should be interpreted as reflecting clinically significant insomnia symptoms rather than a formal DSM-5 diagnosis of insomnia disorder. Although a validated Serbian version of the ISI has not been formally published, the instrument has been widely used in Serbian-speaking populations. Fifth, although participants with self-reported diagnosis of psychiatric disorder(s) were excluded, validated measures of depressive and anxiety symptoms were not included. This represents an important limitation, as subclinical or undiagnosed affective symptoms may have acted as unmeasured confounders, potentially influencing the observed associations. In addition, participants who reported the use of anxiolytic medications were retained in the analysis. These medications may be prescribed for non-psychiatric indications or may be self-initiated. However, it must be noted that anxiolytic use may reflect underlying anxiety or depressive symptoms that were either subclinical or not formally diagnosed. Finally, although multiple relevant variables were included in the analysis, the multivariable model explained a relatively modest proportion of the variance in clinically significant insomnia symptoms, suggesting that additional psychological, environmental, or genetic factors not captured in this study may also contribute to clinically significant insomnia symptoms. Despite these limitations, the study provides important epidemiological evidence on clinically significant insomnia symptoms and their associated factors in a large adult population of Serbia.

## 5. Conclusions

In summary, the presence of clinically significant insomnia symptoms in Serbian adults is influenced by a complex interplay of sociodemographic factors, comorbidities, psychosocial stressors, and lifestyle behaviors. Single or divorced marital status, lower education, student or retired employment status, dermatological comorbidities, anxiolytic use, exposure to stressful events, late-night activities, caffeine and energy drink consumption, and late-night eating were independently associated with higher risk for clinically significant insomnia symptoms. These findings underscore the need for integrated approaches that address medical, psychosocial, and behavioral interventions to prevent and manage insomnia symptomatology. Future longitudinal studies are warranted to further elucidate causal pathways and evaluate the effectiveness of targeted interventions.

## Figures and Tables

**Table 1:** Sociodemographic characteristics of the study population according to insomnia status.

Variables	Total (*n* = 2577)	ISI (≥15) (*n* = 281)	ISI (<15) (*n* = 2296)	*p*-Value
**Sex, *n* (%)** **Male** **Female**	1257 (48.8) 1320 (51.2)	146 (52.0) 135 (48.0)	1111 (48.4) 1185 (51.6)	0.259
**Age, median (range)**	39 (18–89)	35 (18–89)	39 (18–85)	0.052
**Age groups, *n* (%)** **18–29 years** **30–44 years** **45–59 years** **60+ years**	620 (24.1) 953 (37.0) 664 (25.8) 340 (13.2)	95 (33.8) 83 (29.5) 58 (20.6) 45 (16.0)	525 (22.9) 870 (37.9) 606 (26.4) 295 (12.8)	0.043
**Marital status, *n* (%)** **Married** **Single** **Divorced** **Widow/widower**	1284 (49.8) 1021 (39.6) 164 (6.4) 108 (4.2)	106 (37.7) 135 (48.0) 19 (6.8) 21 (7.5)	1178 (51.3) 886 (38.6) 145 (6.3) 87 (3.8)	<0.001
**Children, *n* (%)**	1390 (53.9)	152 (54.1)	1238 (53.9)	0.956
**Region, *n* (%)** **Vojvodina** **Belgrade region** **Šumadija & Western Serbia** **Southern & Eastern Serbia**	752 (29.2) 635 (24.6) 599 (23.2) 591 (22.9)	80 (28.5) 60 (21.4) 87 (31) 54 (19.2)	672 (29.3) 575 (25.0) 512 (22.3) 537 (23.4)	0.010
**Living environment, *n* (%)** **Urban** **Rural**	2047 (79.4) 530 (20.6)	222 (79) 59 (21)	1825 (79.5) 471 (20.5)	0.850
**Residential type, *n* (%)** **House** **Apartment**	1393 (54.1) 1184 (45.9)	163 (58) 118 (42)	1230 (53.6) 1066 (46.4)	0.159
**Educational attainment, *n* (%)** **Primary school** **Secondary school** **Post-secondary/College** **University/PhD**	55 (2.1) 1053 (40.9) 327 (12.7) 1141 (44.3)	11 (3.9) 147 (52.5) 23 (8.2) 99 (35.4)	44 (1.9) 906 (39.5) 304 (13.2) 1042 (45.4)	<0.001
**Employment status, *n* (%)** **Employed** **Unemployed** **Retired** **Student**	1974 (76.6) 166 (6.4) 257 (10.0) 180 (7.0)	187 (66.5) 12 (4.3) 47 (16.7) 35 (12.5)	1787 (77.8) 154 (6.7) 210 (9.1) 145 (6.3)	<0.001

**Table 2:** Distribution of comorbidities, medication use, psychiatric heredity, and stressful life events according to insomnia status.

Variables	Total (*n* = 2577)	ISI (≥15) (*n* = 281)	ISI (<15) (*n* = 2296)	*p*-Value
**Comorbidities, *n* (%)**	473 (18.4)	70 (24.9)	403 (17.6)	0.003
**Cardiovascular comorbidity, *n* (%)**	298 (11.6)	53 (18.9)	245 (10.7)	<0.001
**Rheumatological** **comorbidity, *n* (%)**	37 (1.4)	7 (2.5)	30 (1.3)	0.114
**Respiratory** **comorbidity, *n* (%)**	40 (1.6)	11 (3.9)	29 (1.3)	0.003
**Endocrine and metabolic** **comorbidity, *n* (%)**	153 (5.9)	14 (5.0)	139 (6.1)	0.473
**Gastrointestinal** **comorbidity, *n* (%)**	17 (0.7)	1 (0.4)	16 (0.7)	1000
**Neurological** **comorbidity, *n* (%)**	8 (0.3)	0 (0.0)	8 (0.3)	1000
**Oncological** **comorbidity, *n* (%)**	19 (0.7)	5 (1.8)	14 (0.6)	0.048
**Dermatological** **comorbidity, *n* (%)**	28 (1.1)	8 (2.8)	20 (0.9)	0.008
**Nephrological** **comorbidity, *n* (%)**	4 (0.2)	0 (0.0)	4 (0.2)	1000
**Psychotropic agents, *n* (%)**	170 (6.6)	41 (14.6)	129 (5.6)	<0.001
**Anxiolytic agents, *n* (%)**	158 (6.1)	38 (13.5)	120 (5.2)	<0.001
**Antidepressive agents, *n* (%)**	5 (0.2)	2 (0.7)	3 (0.1)	0.095
**Antiepileptic agents, *n* (%)**	6 (0.2)	0 (0)	6 (0.3)	1000
**Psychiatric heredity, *n* (%)**	158 (6.4)	7 (2.5)	151 (6.9)	0.003
**Stressful events, *n* (%)**	888 (34.5)	137 (48.8)	751 (32.7)	<0.001

**Table 3:** Lifestyle characteristics and dietary habits of the study population according to insomnia status.

Variables	Total (*n* = 2577)	ISI (≥15) (*n* = 281)	ISI (<15) (*n* = 2296)	*p*-Value
**Physical activity, *n* (%)**	741 (28.8)	82 (29.2)	659 (28.7)	0.867
**Late-night activities (TV watching, reading, or hobbies), *n* (%)** **No** **Yes, 2–3× per week** **Yes, every day**	549 (21.3) 894 (34.7) 1134 (44.0)	37 (13.2) 81 (28.8) 163 (58.0)	512 (22.3) 813 (35.4) 971 (42.3)	<0.001
**Smoking, *n* (%)**	924 (35.9)	103 (36.7)	821 (35.8)	0.783
**Alcohol consumption, *n* (%)** **No** **Sometimes, during celebration** **Every weekend** **Everyday**	732 (28.4) 1543 (59.9) 212 (8.2) 90 (3.5)	75 (26.7) 188 (66.9) 8 (2.8) 10 (3.6)	657 (28.6) 1355 (59.0) 204 (8.9) 80 (3.5)	0.460
**Coffee/tea consumption, *n* (%)**	2194 (85.3)	259 (92.2)	1935 (84.3)	<0.001
**Energy drinks consumption, *n* (%)** **No** **Sometimes, 2–3× per week** **Every day**	2219 (86.1) 317 (12.3) 41 (1.6)	221 (78.6) 44 (15.7) 16 (5.7)	1998 (87.0) 273 (11.9) 25 (1.1)	<0.001
**Nutritional habits, *n* (%)** **Regular** **Irregular**	1714(66.5) 863 (33.5)	163 (58.0) 118 (42.0)	1551 (67.6) 745 (32.4)	0.001
**Food in late hours, *n* (%)** **No** **Rarely** **Sometimes, 2–4× per week** **Every day**	518 (20.1) 788 (30.6) 763 (29.6) 508 (19.7)	55 (19.6) 55 (19.6) 96 (34.2) 75 (26.7)	463 (20.2) 733 (31.9) 667 (29.1) 433 (18.9)	0.001

## Data Availability

The data underlying this article will be shared on reasonable request to the corresponding author.

## Abbreviations

The following abbreviations are used in this manuscript:

DSM-5	*Diagnostic and Statistical Manual of Mental Disorders*, Fifth Edition
ISI	Insomnia Severity Index
COVID-19	Coronavirus Disease 2019
SPSS	Statistical Package for the Social Sciences
CI	Confidence Interval
OR	Odds Ratio
GDP	Gross Domestic Product
